# Pre-processing tissue specimens with a tissue homogenizer: clinical and microbiological evaluation

**DOI:** 10.1186/s12866-021-02271-6

**Published:** 2021-07-02

**Authors:** Erlangga Yusuf, Marieke Pronk, Mireille van Westreenen

**Affiliations:** Department of Medical Microbiology and Infectious Diseases, Erasmus MC University Medical Center, Doctor Molewaterplein 40, 3015 GD Rotterdam, the Netherlands

**Keywords:** tissue, tissue homogenizer, culture, microbiological yield

## Abstract

**Background:**

Tissues are valuable specimens in diagnostic microbiology because they are often obtained by invasive methods, and effort should thus be taken to maximize microbiological yield. The objective of this study was to evaluate the added value of using tissue pre-processing (tissue homogenizer instrument gentleMACS Dissociator) in detecting microorganisms responsible for infections.

**Methods:**

We included 104 randomly collected tissue samples, 41 (39.4 %) bones and 63 (60.6 %) soft tissues, many of those (42/104 (40.4 %)) were of periprosthetic origins. We compared the agreement between pre-processing tissues using tissue homogenizer with routine microbiology diagnostic procedure, and we calculated the performance of these methods when clinical infections were used as reference standard.

**Results:**

There was no significant difference between the two methods (McNemar test, *p* = 0.3). Among the positive culture using both methods (*n* = 62), 61 (98.4 %) showed at least one similar microorganism. Exactly similar microorganisms were found in 42/62 (67.7 %) of the samples. From the included tissues, 55/ 104 (52.9 %) were deemed as infected. We found that the sensitivity of homogenized tissue procedure was lower (83.6 %) than when tissue was processed using tissue homogenizer (89.1 %). Sub-analysis on periprosthetic tissues and soft or bone tissues showed comparable results.

**Conclusions:**

The added value of GentleMACS Dissociator tissue homogenizer is limited in comparison to routine tissue processing.

**Supplementary Information:**

The online version contains supplementary material available at 10.1186/s12866-021-02271-6.

## Introduction

Tissues are valuable specimens in diagnostic microbiology because they are often obtained by invasive methods such as synovectomy or osteotomy [1]. Effort should thus be taken to maximize microbiological yield and to prevent contamination. In routine procedure, tissue specimens are often streaked and rolled on solid culture media. Yet, this method does not allow exposition of all parts of the tissue to the surface of the solid culture media. Consequently, this may reduce the detection rate of the microorganisms. To increase the probability of detecting microorganisms, many routine microbiological laboratories also incubate tissue in enrichment media or broth, but this may increase contamination rate.

Tissue homogenizers have been used in research setting to homogenize tissue to increase the release of cells from tissue specimens [2, 3]. Arguably, they may also be used to release the microorganisms attached to the tissues. Additional benefit of tissue homogenizers is a standardized manner of tissue specimen processing. One of the tissue homogenizers available in the market is gentleMACS Dissociator (Miltenyi Biotec GmbH, Bergisch Gladbach, Germany), a benchtop instrument that uses a rotor - stator for the semi-automated dissociation of tissues into single-cell suspensions or thorough homogenates. To the best of our knowledge, no earlier study has evaluated the performance of gentleMACS Dissociator tissue homogenizers in tissues from clinical samples.

The aim of this study is to evaluate the performance of gentleMACS Dissociator in detecting microorganisms responsible for infections in comparison with conventional microbiology diagnostic without tissue homogenizers.

## Materials and methods

### Tissue samples and sample size

Tissue samples were convenient samples, randomly selected from tissues originated from patients admitted to the Erasmus University Medical Center, Rotterdam between October 2017 and April 2020. Only one sample per patient was selected, and from these samples, data on anatomical origin and clinical infection diagnosis were obtained. The study was retrospective in nature using limited demographic data and not subjected to Medical Research Involving Human Subjects Act (waiver was granted by Erasmus MC medical ethical commission (MEC 2015 − 306).

No formal sample size calculation was performed, and we aimed at around 100 samples, in line with previous publications regarding microbiological culture of tissue samples [4–6].

### Microbiological procedure

The samples were processed with gloved hands in a class 2 microbiological safety cabinet. A tissue specimen was brought to a sterile petri dish and cut into several pieces with a diameter around 3mm using sterile scalpel. Determined visually, half part of the tissues was subjected to routine procedure and the other half to pre-processing using gentleMACS Dissociator. The processing and reading of microbiological results were performed by various experienced laboratory technicians, blinded for clinical and the results of other microbiological tests.

In the routine procedure, the following solid agar plates were inoculated by rolling the tissue and printing the tissue into the agar: Columbia with 5 % sheep blood, Chocolate, and MacConkey. The inoculated plates were incubated under aerobic and 5 % CO_2_ atmospheres at 35 °C. The suspension was also seeded on Brucella blood agar plate and incubated under anaerobic condition at 35 °C. All plates were incubated for seven days except for plates of tissues originated from clinical suspicion of prosthetic or osteosynthesis material infection that were incubated for fourteen days. For fungal culture, Sabouraud Glucose Agar was used and incubated at 26 °C, and 35 °C for 21-days. All used agar plates were commercially obtained from BD Diagnostics, Erembodegem, Belgium. No broth cultures of tissue specimens was performed due to often limited amount of tissues.

Growth of microorganism was identified using matrix-assisted laser desorption/ionisation time-of-flight mass spectrometry (Bruker Daltonik, Bremen, Germany) and reported when > 2 colonies on the agar was found.

Gram staining of the tissues was performed using Aerospray® Gram Series 2 Slide Stainer/Cytocentrifuge (ELITech Group, Puteaux, France), and the stained slides were then assessed using an Olympus light microscope with 10x to 100x objective augmentation to assess the presence of microorganisms (cocci or rods) and leukocytes.

### Tissue homogenizing using gentleMACS dissociator

The half part of the tissue was put into a disposable gentleMACS M Tube, and 1.5 ml of sterile physiologic NaCl solution was added. The tube was inserted into the gentleMACS Dissociator and the program RNA_01 was run to homogenize all types of tissue. The duration of this program was optimized at 1 min.

The resulting homogenized suspension then underwent the same routine procedures as mentioned above. The homogenized samples were processed at same time as routine procedure.

### Statistical analysis

We performed two analyses. First, analysis comparing the pre-processing and routine tissue processing results. To this aim, we created 2 × 2 tables and performed McNemar test. Since this comparison may cause circular reasoning (i.e. which method should be used a reference standard), we also performed a second analysis. In this analysis, we used clinical infections as reference standard in order to have fair comparison between the two methods. The clinical infection data were obtained from the patient charts, and evaluated by a clinical microbiologist, who was blinded to the tissue pre-processing information. The clinical infection diagnosis was made using clinicians clinical judgement and standardized criteria. For example for the diagnosis of periprosthetic joint infection or osteomyelitis, the patient should have clinical symptoms and signs (such as pain and swelling) and laboratory findings (such as increasing C-reactive protein) in combination with multiple tissue cultures positive with the same microorganism. For endocarditis, Duke’s criteria was used. We calculated the sensitivity and the specificity with its 95 % confidence interval (95 %CI) of routine and tissue homogenizer procedure by comparing the number of positive tissue cultures with this infection status. We performed the sub-analysis on periprosthetic tissues and for bone and joint tissue. We also performed sensitivity analysis by excluding tissues that were expected to grow microorganisms due to commensals such as tissues from ear, nose, and throat (ENT) and gastrointestinal tracts. Further, we investigated in case of polymicrobial growth, whether tissue pre-processing lead to detection of additional microorganism, and whether this additional microorganism was of clinical importance (i.e. the culture of this additional microorganism would lead to different antibiotics choice). Further, we compared the proportion of positive microorganisms and leukocytes on Gram-staining when the tissue was processed routinely or using tissue homogenizer by using chi-square test. Data were analysed using SPSS Statistics 26 (SPSS Inc., Chicago, Ill, USA).

## Results

### Samples

We included 104 tissue samples (41 (39.4 %) bones and 63 (60.6 %) soft tissues). The tissues were originated mostly from the extremities (*n* = 29/ 104, 27.9 %), and periprosthetic tissues (*n* = 42/104, 40.4 %). Most bone samples were from the extremities (*n* = 21/41, 51.2 %) (Supplementary Table 1). The majority of soft tissues was also obtained from periprosthetic location (*n* = 34/104, 32.7 %).

### Agreement between pre-processing and routine processing

The 2 × 2 table comparing the culture results when tissue pre-processing was performed with routine tissue processing is presented in Table 1. Culture using routine procedure were positive for 69 tissues, 25/69 (36.2 %) of them were polymicrobial (Supplementary Table 1). There was no significant difference between the two methods (*p* = 0.3). In 62 samples growth of any microorganism was found in both methods, and in 61 (98.4 %) of them at least one similar microorganism was found. Exactly similar microorganisms were found in 42/62 (67.7 %) of the samples.

**Table 1:** 2 × 2 table comparing the results of routine tissue processing with pre-processing the tissues

		Routine		
		Any growth	No growth	Total
**Pre-processing using tissue homogenizer**	Any growth	62	2	64
	No growth	7	33	40
		69	35	104

### Comparison of culture performance using clinical infection as reference standard

From the included tissues, 55/ 104 (52.9 %) were originated from clinical infection. The sensitivity of routine procedure was lower (83.6 %) than when tissue was processed using tissue homogenizer (89.1 %) (Table 2). The subgroup analysis including periprosthetic tissues only also showed that the sensitivity using pre-processing tissue method was lower than routine procedure only (96.3 % vs. 77.8 %). While the sensitivity of culture of bone tissues was comparable whether tissue homogenizer was used or not, the culture sensitivity using tissue homogenizers was lower than routine procedure (77.1 % vs. 88.6 %). Further comparison between tissue homogenizers and routine procedure is presented in Table 3.

**Table 2:** Performance of routine culture and pre-processing using tissue homogenizer using clinical infection as reference standard

	Sensitivity (95 %CI)	Specificity (95 %CI)
**All tissues (***n*** = 104)**		
Routine procedure	89.1 (80.9 to 97.3)	61.2 (48.3 to 74.1)
With pre- processing	83.6 (73.8 to 93.3)	63.3 (50.1 to 76.0)
**Periprosthetic tissues only (***n*** = 42)**		
Routine procedure	96.3 (91.3 to 100.0)	93.3 (86.7 to 99.9)
With pre- processing	77.8 (66.8 to 88.8)	93.3 (86.7 to 99.9)
**Bone tissues only (***n*** = 41)**		
Routine procedure	90 (82.1 to 97.9)	71.4 (59.5 to 83.3)
With pre- processing	95 (89.2 to 100.0)	76.2 (64.9 to 87.4)
**Soft tissues only (***n*** = 63)**		
Routine procedure	88.6 (80.2 to 97.0)	53.6 (40.4 to 66.8)
With pre- processing	77.1 (66.0 to 88.2)	53.6 (40.4 to 66.8)
**Only tissues without possible commensals (ENT and GI-tract) (***n*** = 88)**		
Routine procedure	87.8 (79.2 to 96.4)	76.9 (65.8 to 88.0)
With pre- processing	81.6 (71.4 to 100.0)	79.5 (68.8 to 90.2)

Abbreviations: ENT: ear, nose and throat, GI: gastrointestinal

**Table 3:** Additional values of pre-processing tissue

	Total infected tissues, *n* = 55 (%)
Detected exactly the same number and identification of microorganisms as routine	30 (54.5)
Detected monomicrobial that otherwise would be missed	2 (3.6)
Missed monomicrobial that was detected in routine	3 (5.4)
Detected more number of microorganisms of clinical importance in polymicrobial infections	8 (14.5)
Detected more number of microorganisms not of clinical importance in polymicrobial infections	3 (5.4)
Detected less number of microorganisms of clinical importance in polymicrobial infections	8 (14.5)
Detected less number of microorganisms not of clinical importance in polymicrobial infections	1 (1.8)

From the nine tissues with discordant microbiology results (Table 1), in one tissue, the growth of microorganism was deemed as contamination rather than infection (*Cutibacterium acnes* in the tissue from a patient who had tibia fracture). Eight other tissues were deemed as infected. Tissue homogenizer detected one *S. aureus* (endocarditis) and one *E. faecium* (prosthetic joint infection) that would be otherwise missed by routine procedure, but it also missed three coagulase negative *Staphylococci* (two prosthetic joint infection and one low-grade osteosynthesis material infection), two *Serratia marcescens* (both prosthetic joint infection), and one *Staphylococcus aureus* (prosthetic joint infection) which were detected using routine procedures (Supplementary Table 1).

### Comparison of Gram -staining findings

Gram-staining for microorganisms were positive in 16/ 55 (29.1 %) of infected tissues processed using routine procedure and in 14 (25.5 %) using tissue homogenizer. Leukocytes were found in of infected tissues processed routinely in 20/55 (36.4 %) samples in comparison with 15/55 (27.3 %) samples processed using tissue homogenizers (*p* = 0.774 and 0.424, respectively).

## Discussion

There is a clear need to improve the sensitivity of tissue culture, especially in processing periprosthetic tissue [7–10]. Simple direct streaking the tissue on the agar plates may have limited sensitivity. Vortexing the tissue, or culturing the tissues in broth or blood culture bottles have been proposed to increase the yield of the cultures [11, 12]. Yet, these approaches can lead to contamination of the culture. Chemical lysis and automatic tissue homogenizer are other methods that have been proposed to increase the diagnostic sensitivity of microbiological tissue culture [2].

In our study, we found that gentleMACS Dissociator tissue homogenizers (that used rotor- stator method) showed no difference when it was compared with routine microbiological processing of tissues. Yet, using clinical infection as reference standard, it has lower performance. To the best of our knowledge, this is the first study which evaluates the performance of this tissue homogenizer in clinical samples, therefore we cannot make any comparison. The results of our study do show some parallel with the results from an animal study [13]. In that study, *Mycobacterium leprae* was injected to the hind legs of six mice, and after euthanasia, the tissues from the hindleg were obtained and cultured. The results of that study showed that bacteria yield of the tissue processed using gentleMACS Dissociator was lower than without tissue processing [13]. Another study with another tissue homogenizer instrument (MagNA Lyser Rotor, Roche) in pork tissue inoculated with *S. aureus* and *E. coli*, also showed that the number of viable bacteria was reduced compared to control [12]. Next to rotor – stator method, bead mills can be used to homogenize tissue. This bead mills method also performed worse than routine microbiological method, as shown in a study using Ultra-TurrAX bead mills instrument (Axonlab AG) in 38 periprosthetic tissues of patients with knee arthroplasty [2]. We can only hypothesize that the tissue homogenizer had lower yield in comparison to routine procedure because part of the bacteria population were destructed in the tissue during the homogenization process, leading to dead cells, or viable but non culturable state [14] Yet, when a homogenizer is used to release bacteria from swabs, the destruction does not seems to occur, perhaps due to protection of synthetic material (swab) to the bacteria, as shown in a study in a study using Precellys 24 (Bertin Technologies) in swabs inoculated with ATCC bacteria [15]. Yet, swab sample is not a preferred type of samples for periprosthetic joint infection or osteomyelitis [16]. In our protocol, 1.5 mL went in with the tissue, and there is a possibility that dilution of the sample may explain the lower yield of tissue homogenizer. However, by the time the tissue homogenization was completed, the final volume was more concentrated. The dilution effect is thus perhaps not significant. Despite its limited performance, the gentleMACS™ Dissociator was convenient to use. The program took 1 min to run and did not have regular mechanical problems. The hand-on time was also relatively short (< 5 min).

This study used relatively large amount of (periprosthetic) tissues used as it can be seen in relatively narrow confidence interval. Positive tissue culture is one of the criteria in diagnosing prosthetic joint infection [10, 17] and the sensitivity of tissue culture is often limited in low grade prosthetic joint infection,. This study also benefits from the use of various clinical samples that reflect routine practice. Despite these strengths, it should be noticed that this study is not designed to study the performance of pre-processing tissue with other microbiological methods in detecting specific infection such as prosthetic joint infection where sonication can be used to enhance the microbiological culture results [18]. Moreover, the generalizability of the results may be limited in case of low grade prosthetic joint infection, since the included tissues mostly originated from revision surgery in which the clinical suspicion of infection was already high, as can be seen in high sensitivity.

The results presented in this study can help the clinical microbiologists, infectious disease specialists and laboratory manager in interpreting the results and implementing tissue homogenizer in routine clinical microbiology.

## Conclusions

Despite of being convenient to use, gentleMACS Dissociator tissue homogenizer was of limited additional value in comparison to conventional tissue processing in routine clinical samples.

## Supplementary information

**Additional file 1.**

## Data Availability

The datasets during and/or analysed during the current study available from the corresponding author on reasonable request.
